# Histological and Immunohistochemical Biomarkers for Wound Age Estimation in Human Skin: A Systematic Review

**DOI:** 10.7759/cureus.100607

**Published:** 2026-01-02

**Authors:** Anas E Ahmed, Wassal F Aljohani, Akram K Moafa, Faisal A Hattan, Yaser A Altalhi, Nawaf S Alsulamy, Zayad M Albalawi, Feras M Aldhubayi, Abdulwhab A Alatawi, Nawaf K Alqahtani

**Affiliations:** 1 Community Medicine, Jazan University, Jazan, SAU; 2 College of Medicine, Taif University, Taif, SAU; 3 College of Medicine, Jazan University, Jazan, SAU; 4 General Practice, Taif University, Taif, SAU; 5 Pediatrics, Royal Commission Hospital, Yanbu, SAU; 6 College of Medicine, Tabuk University, Tabuk, SAU; 7 College of Medicine, Qassim University, Buraydah, SAU

**Keywords:** angiogenesis, biomarkers, cytokines, extracellular matrix, forensic pathology, histology, immune cells, immunohistochemistry, mast cells, wound age estimation

## Abstract

Accurate estimation of wound age is a critical challenge in forensic pathology, particularly for determining wound vitality and the interval between injury and death. Conventional histology often lacks sensitivity in the early post-traumatic stages, prompting investigation into molecular and immunohistochemical biomarkers. This review synthesizes evidence on histological and immunohistochemical markers used for wound age estimation in human skin, with attention to their applicability across different post-traumatic intervals. Very early wounds, from minutes up to one hour, are best characterized by markers related to haemostasis, mast cell activation, and early cytokine release, including fibronectin, CD62p (P-selectin), factor VIII-related antigen, tumor necrosis factor-alpha (TNF-α), and tryptase. Early wound stages, spanning hours, show transient mast cell accumulation and cytokine expression preceding inflammatory cell infiltration. Intermediate wounds, from one to ten days, are associated with immune cell phenotyping and chemokine expression, such as CD14, CD68, interleukin-8 (IL-8), monocyte chemoattractant protein-1 (MCP-1), and macrophage inflammatory protein-1 alpha (MIP-1α). Late wounds, seven days or more, are marked by indicators of hypoxia response, angiogenesis, cellular stress, and extracellular matrix remodeling, including oxygen-regulated protein 150 (ORP150), ubiquitin, vascular endothelial growth factor (VEGF), matrix metalloproteinases, collagens, and aquaporins. No single marker provides sufficient accuracy across all healing phases, highlighting the need for multimodal approaches that combine morphological assessment with panels of temporally complementary biomarkers. Standardization of methods and additional high-quality human studies are essential to improve the reliability and forensic applicability of wound age estimation.

## Introduction and background

Accurate estimation of wound age is a fundamental yet challenging task in forensic pathology, as it is crucial for reconstructing injury events, determining vitality, and establishing timelines relevant to investigations and judicial proceedings [1]. Distinguishing antemortem from postmortem injuries and estimating the time since wounding can influence interpretations of cause and manner of death and support or refute investigative hypotheses [2,3]. However, wound age estimation is complicated by the dynamic and multifactorial nature of tissue responses, interindividual variability, and postmortem changes [4].

Traditionally, estimation has relied on macroscopic examination and routine histopathology [5]. Classical histological features, such as hemorrhage, edema, inflammatory cell infiltration, and fibroblast proliferation, provide a general framework for assessing wound progression but often lack sensitivity and specificity, particularly in the early post-traumatic period [6]. Overlapping patterns across time intervals and the influence of postmortem changes and comorbidities further limit the reliability of conventional histology [7].

Recent advances in molecular biology and immunohistochemistry (IHC) have expanded the tools available to forensic pathologists [8]. Temporal expression of cellular markers, cytokines, growth factors, and stress-response proteins can provide more objective indicators of wound vitality and age [9]. Immunohistochemical techniques allow visualization of specific molecules involved in inflammation, angiogenesis, and tissue repair, helping define early, intermediate, and late phases of healing [10-15].

Despite these advances, the forensic application of these markers remains heterogeneous. Variations in study design, sample selection, wound types, analytical methods, and scoring systems, combined with methodological limitations, hinder the establishment of standardized approaches. No single marker or panel has been universally adopted, highlighting the need for systematic evaluation of existing evidence.

This systematic review aims to critically synthesize current evidence on histological and immunohistochemical markers for wound age estimation, assess their methodological quality, and clarify their diagnostic value, temporal reliability, and practical applicability in forensic practice.

## Review

Methodology

Literature Search Strategy

This systematic review followed the Preferred Reporting Items for Systematic Reviews and Meta-Analyses (PRISMA) guidelines [16]. A comprehensive search was conducted across PubMed, Scopus, Web of Science, and the Cochrane Library from inception to the final search date. The search combined keywords and controlled vocabulary related to wound healing, forensic context, histological and immunohistochemical markers, and wound age. Terms included cytokines, chemokines, mast cell markers, leukocyte antigens, growth factors, coagulation-related proteins, and stress-response proteins. Searches were limited to human studies and original research, excluding reviews, editorials, conference abstracts without data, and non-relevant publications. Database-specific strategies ensured conceptual consistency across platforms (see Appendix 1).

Eligibility Criteria

Studies were included if they examined human skin wounds in a forensic context and assessed histological or immunohistochemical markers to estimate wound age or vitality. Included studies required known or reliably documented wound age and original data linking marker expression to post-traumatic intervals. Early, intermediate, and late wound phases were eligible. Excluded were animal-only studies, studies without forensic relevance, investigations not involving skin, studies limited to macroscopic or routine histology without marker analysis, and publications lacking sufficient methodological detail or outcomes. Non-English studies and case reports were also excluded. No restrictions were applied for geographic location or publication year.

Study Selection

All retrieved records were imported into reference management software, and duplicates were removed. Two reviewers independently screened titles and abstracts to exclude irrelevant studies, followed by full-text assessment against inclusion criteria. Discrepancies were resolved through discussion or consultation with a third reviewer. The selection process was documented using a PRISMA flow diagram, with reasons for exclusion recorded.

Data Extraction and Quality Appraisal

Two reviewers independently extracted data using a standardized form, including study design, forensic setting, sample characteristics, wound type and age, markers evaluated, analytical methods, scoring approaches, and key findings. Information on postmortem interval (PMI), control tissue use, and methodological limitations was also collected. Study quality and risk of bias were assessed using an adapted Quality Assessment of Diagnostic Accuracy Studies-2 (QUADAS-2) tool covering case selection, marker assessment, wound age determination, and flow/timing [17]. Each domain was rated as low, high, or unclear risk of bias, with justifications recorded. Discrepancies were resolved through discussion, and results informed the interpretation of the findings.

Results

Study Selection

**Figure 1:**
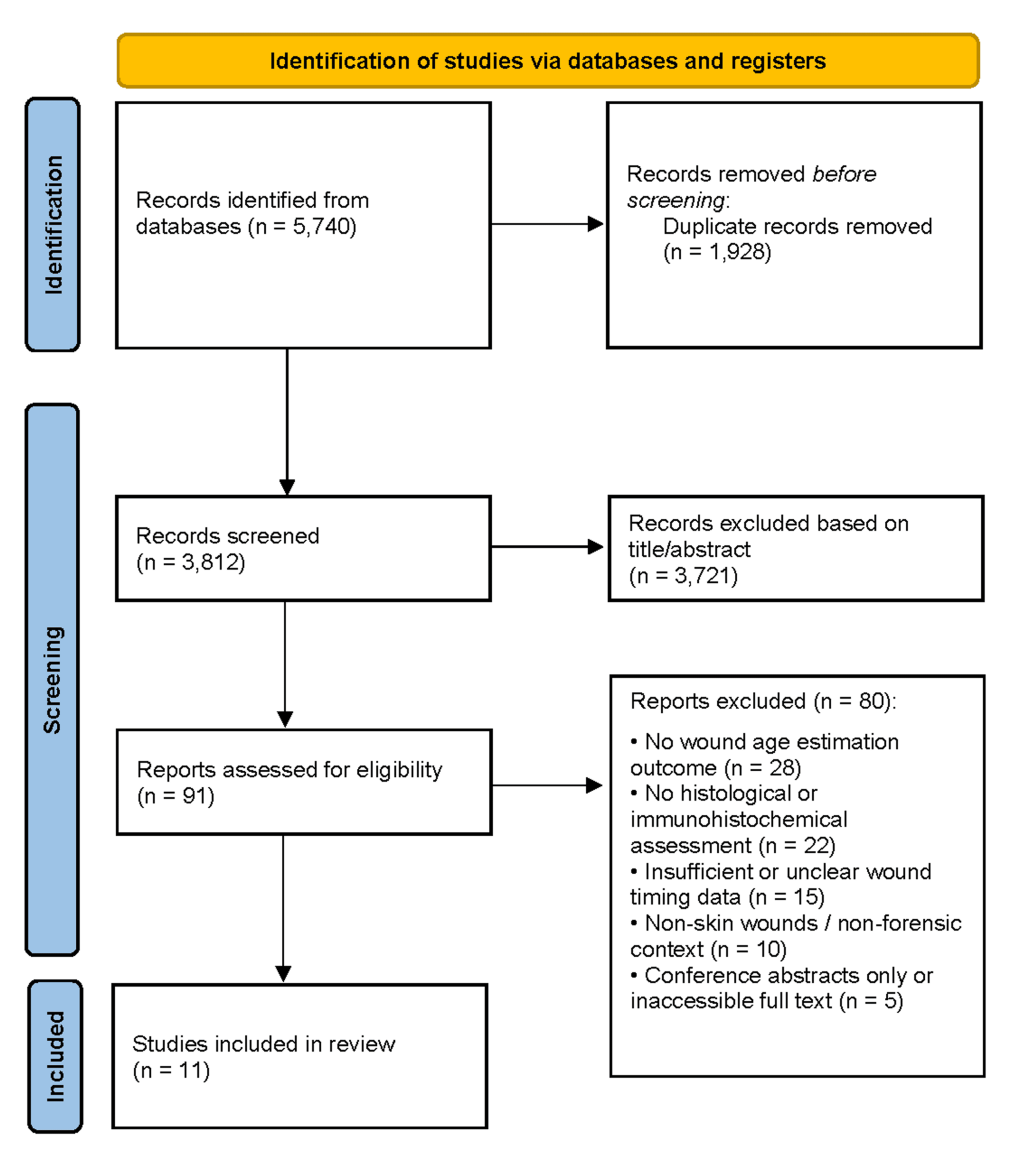
PRISMA flow diagram illustrating the study selection process PRISMA flow diagram illustrating the identification, screening, eligibility assessment, and inclusion of studies in the systematic review, including records retrieved through database searching, duplicates removed, full-text articles assessed for eligibility, and studies included in the final qualitative synthesis [16].

A total of 5,740 records were identified, yielding 3,812 unique records after duplicate removal. Title and abstract screening excluded 3,721 records, primarily due to lack of forensic relevance, absence of wound age estimation, non-skin wounds, non-histological or non-immunohistochemical methods, animal-only studies, or non-original publication types. Ninety-one full-text articles were assessed, of which 80 were excluded for reasons including absence of a forensic context, lack of relevant markers, unclear wound timing, non-skin tissues, or unavailable data. Eleven studies [1-11] met all eligibility criteria and were included in the qualitative synthesis and risk-of-bias assessment (Figure 1).

Study Characteristics

Most included studies were human autopsy-based observational investigations, commonly using non-injured skin as internal controls; one study involved living subjects with known wound ages. Sample sizes ranged from several dozen to several hundred specimens. Wound types included stab and incised wounds, lacerations, abrasions, contusions, surgical incisions, and blunt-force injuries. The temporal scope spanned minutes to several weeks post-injury, with studies typically categorizing wounds into predefined time intervals. Immunohistochemistry on paraffin-embedded sections was the principal method, assessing a broad range of inflammatory mediators, mast cell-related proteins, growth factors, stress-response proteins, and coagulation or extracellular matrix components using semi-quantitative, morphometric, or probability-based analyses (Table 1).

**Table 1:** Summary of characteristics of the included studies Summary of studies evaluating histological and immunohistochemical markers for wound age estimation in human and experimental settings. HPF - high-power field; IHC - immunohistochemistry; Se - sensitivity; Sp - specificity; AUC - area under the receiver operating characteristic curve; MPO - myeloperoxidase; CML - carboxymethyl-lysine; ECM - extracellular matrix; ORP150 - oxygen-regulated protein 150; α-SMA - alpha–smooth muscle actin; TGF-α/β1 - transforming growth factor alpha/beta 1; TNF-α - tumor necrosis factor alpha; IL-1β/IL-6/IL-8 - interleukins 1 beta, 6, and 8; MCP-1 - monocyte chemoattractant protein-1; MIP-1α - macrophage inflammatory protein-1 alpha; CD markers - cluster of differentiation cell surface antigens; FVIIIra - factor VIII-related antigen; Ub - ubiquitin; CD62p - P-selectin; APAAP - alkaline phosphatase-anti-alkaline phosphatase; LSAB - labeled streptavidin-biotin; ↑- increase; ↓ - decrease Wound ages are reported in minutes (min), hours (h), or days (d) post-injury.

Study (author)	Study design and population	Sample size and wound type	Wound age range and groups	Marker(s) and category	Method and quantification	Time-dependent expression	Diagnostic performance	Forensic application	Key findings and limitations
van de Goot et al. [1]	Human autopsy observational study	807 samples; blunt-force; paired controls	Early wounds only; controls, few minutes, 15–30 min	Fibronectin, CD62p, Factor VIII	IHC; 4-grade scoring; probability model	↑within minutes; peak 15–30 min; ↓not assessed	Probability: 0→non-vital 87–90%; 1–2→few min 72–93%; 3→15–30 min 55–76%	Very early wound age (minutes–30 min)	Coagulation markers allow early estimation; limitations: relies on reported wound timing
Bonelli et al. [2]	Human autopsy study with surgical controls	105 skin samples; 75 vital, 15 post-mortem, 15 surgical controls	≤24 h; ≤1, 1–3, 3–6, 6–12, 12–24 h	Mast cells (tryptase/chymase)	Affinity cytochemistry + IHC; quantitative counts	↑detectable ≤1 h; peak 1–3 h; ↓after 3 h	Statistically significant (p<0.01); no ROC	Early wound age (hours), vitality assessment	Transient dermal mast cell increase; limitation: group-based inference, no ROC metrics, reduced utility >24 h
Fronczek et al. [3]	Human study, living subjects	101 wound biopsies; mixed traumatic injuries	0.2–25 d; 0.2–2, 2–4, 4–10, 10–25 d	MPO, CD45, CD68 (cells); IL-8, MIP-1 (chemokines); CML, vitronectin	IHC; cell counts, probability scoring	peak 0.2–2 d; ↓4–10 d; mild secondary ↑10–25 d	>10 IL-8/MIP-1 cells/mm²→<10 d; probability model; no ROC	Early–intermediate wound age (hours–days)	Combined markers improve estimation; limitations: living subjects, overlap, no ROC metrics
Fouad et al. [4]	Human autopsy observational study	40 autopsy cases; stab 55%, laceration 20%, incised 10%, abrasion 10%, contusion 5%	<12 h–10 d; <12 h, 12–24 h, 1–3 d, >3–10 d	CD14 (inflammatory cell surface receptor)	IHC (avidin–biotin); semi-quantitative % CD14+ cells	↑<12 h; peak 1–3 d; ↓>3 d	ROC >90%: 1–3 d (Se/Sp 100%, AUC 1.0); 70% cut-off poor	Intermediate wound age (1–3 d), vitality assessment	Strong correlation with wound age; limitation: small sample in older wounds, limited wound types
Bacci et al. [5]	Human autopsy study with surgical controls	50 vital wounds, 10 post-mortem lesions, 10 surgical controls	≤60 min; ≤5, 6–15, 16–30, 31–60 min	TNF-α–positive mast cells (cytokine)	Affinity cytochemistry + IHC; morphometric counts (cells/mm²)	↑TNF-α >15 min; peak 31–60 min; ↓post-mortem lesions	Statistically significant differences; no sensitivity/specificity	Very early wound age (minutes–1 h), vitality assessment	TNF-α mast cells increase earlier than total mast cells; limitation: short time window, no diagnostic metrics
Ishida et al. [6]	Human autopsy observational study	58 wounds; paired controls	Few minutes–21 d; 0–12 h, 1–5, 7–14, 17–21 d	ORP150 (hypoxia/angiogenesis)	IHC; double-color fluorescence; morphometry	↑≤12 h minimal; peak 7–14 d (~59%); ↓17–21 d (~28%)	Cut-off >50%→7–14 d; no ROC	Intermediate–late wound age (days–weeks)	Reflects hypoxia-driven granulation; limitations: overlap 1–5 and 17–21 d, semi-quantitative
Gauchotte et al. [7]	Human autopsy/surgical study; ex vivo model	12 stab wounds, 58 surgical, 8 controls, 24 early post-mortem	1–44 min; <10, 10–20, >20–44 min	FVIIIra, CD15, mast cell tryptase	IHC on paraffin; quantitative/qualitative	Tryptase degranulation 1 min; CD15 9 min; peak first hour; ↓putrefied tissue	FVIIIra: Se 100%, Sp 47%; CD15: Se 47%, Sp 100%; tryptase: Se 60%, Sp 100%	Very early wound age (minutes), vitality	CD15 and tryptase are reliable early markers; limitations: putrefied tissue, moderate inter-observer agreement
Kondo et al. [8]	Experimental animal study; human validation	55 human wounds; paired controls	Few minutes–21 d; 0–12 h, 1–5, 7–14, 17–21 d	Ubiquitin (Ub, stress/heat-shock)	IHC; semi-quantitative morphometry; blinded observers	↑4 h–1 d; peak 7–14 d (~31–45%); ↓17–21 d, shift to fibroblasts	<10%→<1 d; >10%→≥1 d; >20%→7–14 d; >30%→7–14 d; no ROC	Intermediate to late wound age	Clear cell-type pattern; limitations: overlap 1–5 and 17–21 d, semi-quantitative
Grellner et al. [9]	Human autopsy/surgical study	105 sharp-force wounds; paired controls	Minutes–6 weeks; early: 15–20, 30–60, 60–90 min	IL-1β, IL-6, TNF-α	IHC (APAAP); semi-quantitative	Earliest 15–20 min; peak 30–90 min; ↓several hours	Not reported	Very early wound age (minutes–hours), vitality	Rapid cytokine up-regulation precedes leukocytes; limitations: baseline expression, semi-quantitative
Grellner et al. [10]	Human autopsy/surgical study	74 wounds TGF-α, 51 TGF-β1; paired controls	Minutes–6 weeks; early 5 min–5 h	TGF-α, TGF-β1 (growth factors)	IHC (LSAB); semi-quantitative epidermis/dermis	TGF-α ↑~10 min; TGF-β1 minutes; peak 30–60 min; ↓TGF-α 1–1.5 h; TGF-β1 persists hours–weeks	Not reported	Very early to intermediate/late wound age	Rapid up-regulation precedes inflammation; limitations: baseline expression, semi-quantitative
Kondo et al. [11]	Human autopsy observational study	50 wounds; paired controls	Few minutes–21 d; 0–12 h, 1–4, 7–14, 17–21 d	IL-8, MCP-1, MIP-1α (chemokines)	IHC; semi-quantitative morphometry; two observers	↑4–12 h; peak 1–4 d; ↓7–21 d, but higher than baseline	Cut-offs: IL-8 >50%, MCP-1 >30%, MIP-1α >40%→≥1 d; no ROC	Early–intermediate wound age (≥1 d)	Chemokine shift from neutrophils → macrophages/fibroblasts; limitations: baseline expression, overlap, semi-quantitative

Quality Assessment of Included Studies

Using a tailored QUADAS-2 approach, case selection and reference standards were judged as low risk of bias across all studies, supported by clear inclusion criteria and independent wound timing sources. Variability was observed in the index test domain, with some earlier studies rated as unclear due to limited reporting on blinding or diagnostic thresholds, whereas more recent studies employed standardized scoring and explicit cut-offs. Flow and timing were generally low risk, with one study showing uncertainty due to reliance on witness-reported timing. Overall, most studies were rated as low risk of bias, with a minority classified as moderate quality due to index test reporting limitations (Table 2).

**Table 2:** Summary of methodological quality assessment of included studies Risk-of-bias assessment of studies evaluating histological and immunohistochemical markers for wound age estimation using the QUADAS-2 tool [17]. Case selection refers to the process of defining inclusion and exclusion criteria and the appropriateness of the study population. Index test refers to the immunohistochemical or other marker-based assessment under investigation. The reference standard indicates the independent method used to determine wound age or vitality. Flow and timing refer to uniform sample processing, predefined wound-age groups, and completeness of analysis. Overall risk of bias reflects the combination of all domains, categorized as low, moderate, or high. IHC - immunohistochemistry; TNF-α - tumor necrosis factor alpha; ORP150 - oxygen-regulated protein 150; Ub - ubiquitin; HPF - high-power field; PMI - post-mortem interval; ROC - receiver operating characteristic; APAAP - alkaline phosphatase-anti-alkaline phosphatase

Study (author)	Case selection	Index test	Reference standard	Flow and timing	Overall risk of bias
van de Goot et al. [1]	Low: Large autopsy series (n=322) with clear grouping of controls, very early, and early vital wounds.	Low: IHC scoring system defined; consensus scoring by two assessors.	Low: Wound age based on forensic investigation and witness statements.	Unclear: Reliance on witness statements may introduce timing uncertainty.	Moderate
Bonelli et al. [2]	Low: Clearly defined groups including vital lesions with documented survival times, post-mortem lesions, and healthy surgical controls; no inappropriate exclusions.	Unclear: Affinity cytochemistry and immunofluorescence methods for mast cells are described, but blinding and pre-specified diagnostic cut-offs are not reported.	Low: Wound age established independently from medical, police, and circumstantial records.	Low: All samples processed uniformly, PMI reported, all predefined groups included.	Moderate
Fronczek et al. [3]	Low: Living subjects with known wound age prospectively included; exclusions justified; wound ages documented by victims and forensic physicians.	Low: IHC protocols, antibodies, scoring, and statistical modelling pre-specified; standardized assessment across markers.	Low: Wound age determined independently from patient history and forensic documentation.	Low: Biopsies processed uniformly; wound-age groups predefined; all cases analyzed.	Low
Fouad et al. [4]	Low: Randomly selected autopsy cases with clearly defined inclusion/exclusion criteria; wounds of known infliction time included; paired non-injured skin controls.	Low: IHC staining, antibody details, semi-quantitative assessment, and ROC-based cut-offs are clearly described, reducing interpretation bias.	Low: Wound age independently established from police reports and documented infliction times.	Low: Samples processed uniformly; putrefied cases excluded; PMI ≤3 days; all cases analyzed.	Low
Bacci et al. [5]	Low: Vital wounds, post-mortem lesions, and healthy skin controls are clearly defined; selected based on documented survival times with no inappropriate exclusions.	Unclear: IHC methods and quantification of TNF-α-positive mast cells are described, but blinding and pre-specified cut-offs are not reported.	Low: Wound age independently determined using police, witness, and medical records.	Low: All samples processed uniformly, post-mortem intervals reported, no unexplained exclusions.	Moderate
Ishida et al. [6]	Low: Autopsy skin wounds with defined wound-age groups (0–12 h to 17–21 d); paired uninjured skin controls; confounding conditions excluded.	Low: IHC and morphometric quantification described; two investigators blinded; cut-offs biologically/statistically supported.	Low: Wound age determined independently from forensic documentation; not inferred from ORP150.	Low: Samples processed uniformly; PMI <3 days; predefined groups fully analyzed; no unexplained exclusions.	Low
Gauchotte [7]	Low: Autopsy wounds, surgical intravital wounds, and post-mortem controls included; criteria clearly reported; no inappropriate enrichment.	Low: IHC assessment predefined with explicit thresholds (CD15 ≥4 cells/10 HPF; tryptase degranulation ≥20% + gradient); observers blinded; reproducibility assessed.	Low: Vitality defined using clear histological criteria; ex vivo post-mortem model strengthens reference validity.	Low: All samples underwent the same index tests; timing between injury, devascularization, and sampling documented.	Low
Kondo et al. [8]	Low: 55 human skin wounds across defined age groups (0–12 h to 17–21 d) with paired controls; confounders excluded.	Low: IHC protocol and morphometric quantification clearly described; two blinded investigators; intranuclear Ub positivity assessed.	Low: Wound age independently established using forensic documentation and surgical/autopsy timing.	Low: Samples processed uniformly; PMI controlled; predefined groups fully analyzed; no unexplained exclusions.	Low
Grellner et al. [9]	Low: Large series of human skin wounds with clearly defined wound-age groups; paired uninjured skin controls; no inappropriate exclusions.	Unclear: IHC (APAAP) and semi-quantitative scoring described; blinding and pre-specified cut-offs not reported.	Low: Wound age determined independently from surgical timing and forensic documentation.	Low: Samples processed uniformly; early/late intervals predefined; PMI reported; no unexplained exclusions.	Moderate
Grellner et al. [10]	Low: Skin wounds from operations and autopsies chronologically dated; clear inclusion/exclusion; paired uninjured skin controls.	Low: IHC methods clearly described; assessors blinded; scoring criteria predefined; positive/negative controls used.	Low: Wound age determined independently from surgical records and forensic documentation.	Low: Samples processed uniformly; early/late intervals predefined; PMI reported; no unexplained exclusions.	Low
Kondo et al. [11]	Low: 50 human autopsy skin wounds across defined age groups (0–12 h; 1–4 d; 7–14 d; 17–21 d) with paired controls; confounders excluded.	Low: IHC protocols and morphometric quantification described; two independent investigators; biologically meaningful cut-offs proposed.	Low: Wound age independently established using forensic documentation and autopsy timing.	Low: Uniform processing; PMI <3 days; predefined groups fully analyzed; no unexplained exclusions.	Low

Qualitative Synthesis of Results

Very early wound age assessment demonstrated that immunohistochemical markers detect molecular changes within minutes, preceding histological inflammation. TNF-α-positive mast cells increased as early as 15 minutes and rose with survival time, distinguishing vital from post-mortem lesions [5]. Mast cell degranulation and early granulocyte markers were detectable within minutes, while coagulation-related markers showed rapid up-regulation with peak expression at short survival intervals, favoring vitality in probability-based models [1,7].

Early wound age within hours was characterized by transient mast cell increases peaking within the first few hours, followed by a decline, with consistently lower counts in post-mortem lesions [2]. Pro-inflammatory cytokines, including IL-1β, IL-6, and TNF-α, were up-regulated within minutes and peaked within the first 1-2 hours, providing temporal resolution before cellular infiltration [9].

Intermediate wound age was marked by evolving inflammatory infiltrates and early remodeling. CD14 expression increased within hours, peaked at 1-3 days, and declined thereafter, showing excellent discrimination for wounds aged 1-3 days [4]. Neutrophils peaked earlier than macrophages, while chemokines declined progressively; combined-marker models improved estimation for wounds younger than 10 days [3].

Late wound age estimation relied on stress-response and remodeling markers. Hypoxia-regulated proteins and ubiquitin showed minimal early expression, increased after one day, and peaked at 7-14 days before declining, with cell-type shifts reflecting granulation tissue development. Chemokines followed phase-dependent patterns with reduced but persistent expression in later wounds [6,8].

Discussion

This systematic review synthesizes histological and immunohistochemical markers for wound age estimation using a time-oriented framework that reflects the biological progression of wound healing. The findings confirm that wound repair begins immediately after injury and evolves through overlapping phases characterized by distinct cellular and molecular responses. Conventional histology alone shows limited sensitivity in the earliest post-traumatic period, whereas immunohistochemical markers related to haemostasis, inflammation, and immune activation provide improved temporal resolution. In particular, early expression of coagulation-related proteins, inflammatory cytokines, and mast cell activation markers appears valuable for distinguishing vital from non-vital wounds shortly after injury. As healing progresses, phase-specific immune cell infiltration and mediator expression allow more reliable differentiation of intermediate wound ages, especially when multiple markers are evaluated together rather than in isolation.

For later stages of wound healing, markers associated with angiogenesis, extracellular matrix remodeling, hypoxic stress, and fibroblast activity show more consistent patterns, although increasing biological variability limits precise dating. Across all time intervals, the review highlights that no single marker can reliably estimate wound age, emphasizing the importance of a multimodal approach integrating morphology with panels of complementary immunohistochemical markers. Interpretation remains constrained by methodological heterogeneity, lack of standardized scoring systems, and variability related to individual healing capacity and post-mortem factors. Future research should focus on well-characterized human studies with standardized protocols and validated cut-offs to improve forensic reliability and facilitate routine application in medicolegal practice.

Limitations

This review is limited by substantial methodological heterogeneity among included studies, including differences in wound types, sampling protocols, antibodies, scoring systems, and post-mortem intervals, which prevented quantitative meta-analysis. Many studies relied on semi-quantitative or observer-dependent assessments with limited reporting of diagnostic accuracy. Confounding factors such as comorbidities and environmental conditions were inconsistently addressed. Emerging molecular techniques, while promising, lacked standardization, limiting their applicability. These factors underscore the need for future research using harmonized methodologies and larger, well-characterized human datasets.

## Conclusions

Wound age estimation remains a complex forensic challenge, especially in the early post-traumatic period, where conventional histology has limited sensitivity. Wound healing follows reproducible, phase-dependent molecular and cellular patterns that can guide temporal assessment. Early markers of haemostasis, mast cell activation, and cytokine release are informative within minutes to hours; immune cell and chemokine profiles provide resolution during intermediate phases; and markers of hypoxia response, angiogenesis, and tissue remodeling support dating in later stages. No single biomarker is sufficient across all phases, highlighting the value of integrated marker panels combined with morphological evaluation. Standardized protocols, digital quantification, and validation in human forensic material are essential to enhance accuracy, reproducibility, and reliability in wound age determination.

## Appendices

Appendix 1: summary of search strategy

Pubmed - 1,420: (("Wound Healing"[Mesh] OR "Wounds and Injuries"[Mesh] OR wound* OR lesion*) AND ("Age Determination by Immunologic Techniques"[Mesh] OR immunohistochem* OR histology OR biomarker* OR cytokine* OR chemokine* OR "mast cell*" OR CD14 OR CD15 OR tryptase OR IL-1 OR IL-6 OR TNF-alpha OR TGF-beta OR ORP150 OR ubiquitin) AND ("Forensic Medicine"[Mesh] OR forensic* OR medicolegal OR autopsy OR postmortem) AND ("wound age" OR "age of wound" OR "timing of wounds" OR "survival time" OR "post-traumatic interval") )

Cochrane - 186: ("wound age" OR "timing wounds") AND (forensic* OR medicolegal) AND (immunohistochem* OR histolog* OR biomarker*)

Scopus - 2345: TITLE-ABS-KEY( (wound* OR lesion*) AND (forensic* OR medicolegal OR autopsy OR postmortem) AND (histolog* OR immunohistochem* OR biomarker* OR cytokine* OR chemokine* OR "mast cell*" OR CD14 OR CD15 OR tryptase OR IL-1 OR IL-6 OR TNF-alpha OR TGF-beta OR ubiquitin) AND ("wound age" OR "age of wound" OR "wound aging" OR "post-traumatic interval" OR "survival time"))

WOS - 1789: TS=((wound* OR lesion*) AND (forensic* OR medicolegal OR autopsy OR postmortem) AND (histolog* OR immunohistochem* OR biomarker* OR cytokine* OR chemokine* OR "mast cell*" OR CD14 OR CD15 OR tryptase OR IL-1 OR IL-6 OR TNF-alpha OR TGF-beta OR ubiquitin) AND ("wound age" OR "age of wound" OR "timing wound*" OR "post-traumatic interval" OR "survival time"))

## References

[1] van de Goot FR, Korkmaz HI, Fronczek J (2014). A new method to determine wound age in early vital skin injuries: a probability scoring system using expression levels of Fibronectin, CD62p and Factor VIII in wound hemorrhage. Forensic Sci Int.

[2] Bonelli A, Bacci S, Norelli GA (2003). Affinity cytochemistry analysis of mast cells in skin lesions: a possible tool to assess the timing of lesions after death. Int J Legal Med.

[3] Fronczek J, Lulf R, Korkmaz HI (2015). Analysis of inflammatory cells and mediators in skin wound biopsies to determine wound age in living subjects in forensic medicine. Forensic Sci Int.

[4] Fouad AA, Badr El Dine MMF, El Dine Menesy MKH, Abdelatif AA, Abdelatif AA, Khedr IR (2019). Detection of the timing of human skin wounds by immunohistochemical analysis of CD14. Arab J Forensic Sci Forensic Med.

[5] Bacci S, Romagnoli P, Norelli GA, Forestieri AL, Bonelli A (2006). Early increase in TNF-alpha-containing mast cells in skin lesions. Int J Legal Med.

[6] Ishida Y, Kimura A, Takayasu T, Eisenmenger W, Kondo T (2008). Expression of oxygen-regulated protein 150 (ORP150) in skin wound healing and its application for wound age determination. Int J Legal Med.

[7] Gauchotte G, Wissler MP, Casse JM (2013). FVIIIra, CD15, and tryptase performance in the diagnosis of skin stab wound vitality in forensic pathology. Int J Legal Med.

[8] Kondo T, Ohshima T, Mori R, Guan DW, Ohshima K, Eisenmenger W (2002). Immunohistochemical detection of chemokines in human skin wounds and its application to wound age determination. Int J Legal Med.

[9] Grellner W (2002). Time-dependent immunohistochemical detection of proinflammatory cytokines (IL-1beta, IL-6, TNF-alpha) in human skin wounds. Forensic Sci Int.

[10] Grellner W, Vieler S, Madea B (2005). Transforming growth factors (TGF-alpha and TGF-beta1) in the determination of vitality and wound age: immunohistochemical study on human skin wounds. Forensic Sci Int.

[11] Kondo T, Tanaka J, Ishida Y, Mori R, Takayasu T, Ohshima T (2002). Ubiquitin expression in skin wounds and its application to forensic wound age determination. Int J Legal Med.

[12] Khalid KA, Nawi AF, Zulkifli N, Barkat MA, Hadi H (2022). Aging and wound healing of the skin: a review of clinical and pathophysiological hallmarks. Life.

[13] Kuninaka Y, Ishida Y, Ishigami A (2022). Macrophage polarity and wound age determination. Sci Rep.

[14] Li N, Du Q, Bai R, Sun J (2020). Vitality and wound-age estimation in forensic pathology: review and future prospects. Forensic Sci Res.

[15] Ren K, Wang L, Wang Y (2022). Wound age estimation based on next-generation sequencing: Fitting the optimal index system using machine learning. Forensic Sci Int Genet.

[16] Page MJ, Moher D, Bossuyt PM (2021). PRISMA 2020 explanation and elaboration: updated guidance and exemplars for reporting systematic reviews. BMJ.

[17] Whiting PF, Rutjes AW, Westwood ME (2011). QUADAS-2: a revised tool for the quality assessment of diagnostic accuracy studies. Ann Intern Med.

